# Normal Human Lactation: closing the gap

**DOI:** 10.12688/f1000research.14452.1

**Published:** 2018-06-20

**Authors:** Melinda Boss, Hazel Gardner, Peter Hartmann

**Affiliations:** 1 M315 School of Allied Health, Faculty of Health and Medical Sciences, University of Western Australia, Crawley, Australia; 2 M313 School of Molecular Sciences, Faculty of Science, University of Western Australia, Crawley, Australia

**Keywords:** Lactation, Breastfeeding, Reference ranges, Reference limits, Normal function, Maternal, Breastmilk

## Abstract

With the exception of infant growth, there are no well-defined parameters describing normal human lactation. This represents a major gap in the continuum of care that does not exist for other major organs. Biological normality occurs naturally and is characterized by well-integrated function. We have proposed a definition that highlights four key elements that describe parameters for biological normality: comfort, milk supply, infant health, and maternal health. Notwithstanding the current limitations, published data have been collated to provide preliminary markers for the initiation of lactation and to describe objective tests once lactation is established. Reference limits have been calculated for maternal markers of secretory activation, including progesterone in maternal blood and total protein, lactose, sodium, and citrate in maternal milk. Objective measurements for established lactation, including 3-hourly pumping and 24-hour milk production, together with pre-feed to post-feed milk fat changes (a useful indicator of the available milk removed by the infant) have been outlined. Considered together with the parameters describing normal function, this information provides a preliminary objective framework for the assessment of human lactation.

## Introduction

The rationale for writing a review is usually based on bringing together recent major advances and published discussion in a particular area of research. Unfortunately, basic research into the physiology and biochemistry of the lactating human mammary gland is limited, and there have been no major advances toward the assessment of its normal function in recent times. As a result, the lactating mammary gland is a poor cousin when compared with other major organs such as the heart, brain, liver, lungs, and kidneys. These all have an array of objective tests available to assess function. This review examines the evidence available toward the development of methods for the objective assessment of lactation. Discussion will be limited to the maternal aspects of lactation physiology and biochemistry during the period of exclusive breastfeeding of the infant (that is, the period of time from birth and during the period of exclusive breastfeeding).

**Figure 1.**
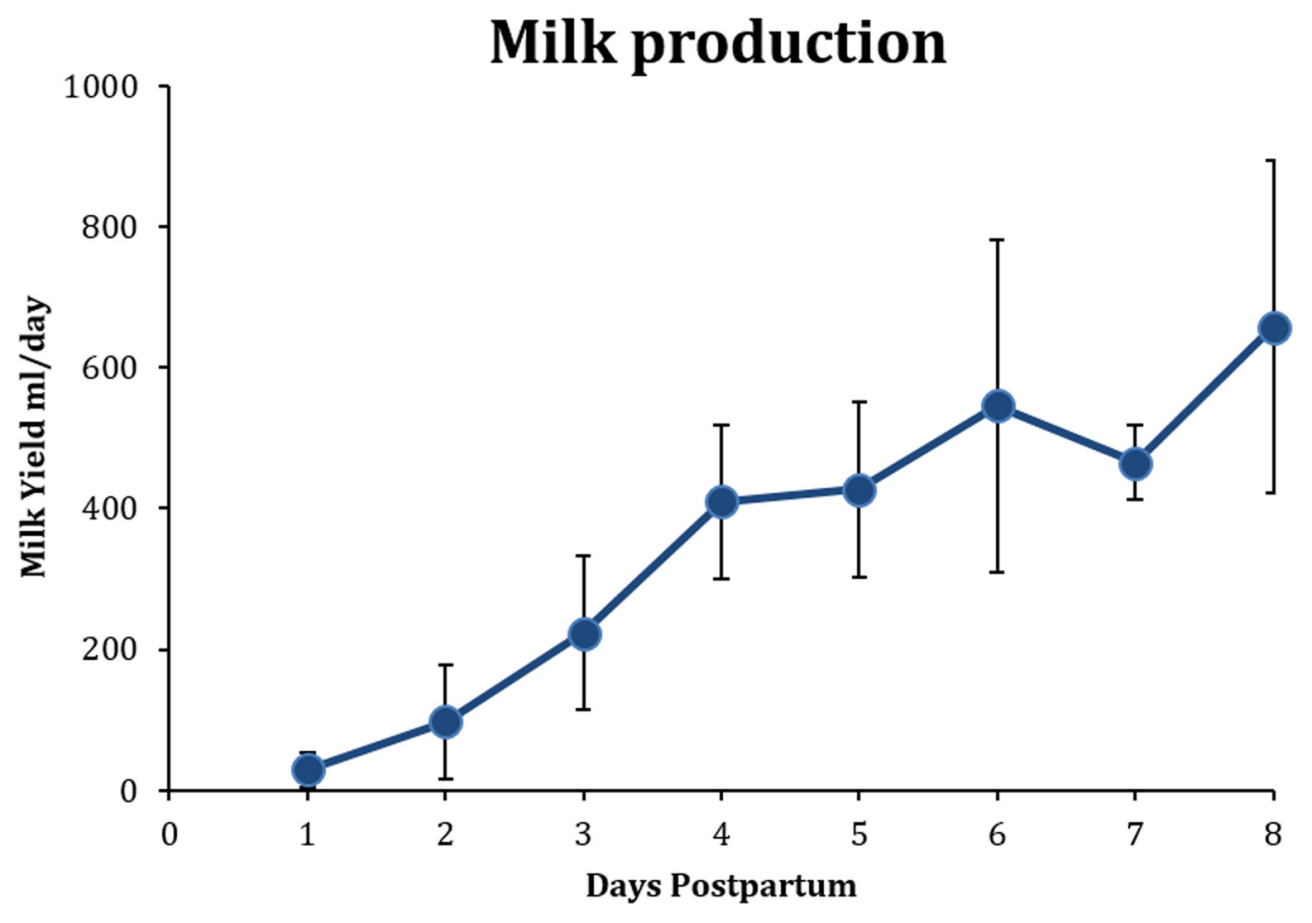
Daily values for milk production in the first 8 days postpartum. Figure shows weighted means and standard deviations collated from published values. Data sources: 5– 14.

The mean milk production of lactating women by 8 days postpartum ( Figure 1) is 650 mL/24 hours, and from 1 to 6 months of lactation the mean range for exclusively breastfed infants is between 750 and 800 mL/24 hours ^1^. Therefore, for mothers exclusively breastfeeding their babies, it can be calculated that the energy output in human milk accounts for about 20% to 30% of the maternal resting energy requirement ^2^. This large energy commitment clearly demonstrates the evolutionary importance of lactation for human survival. The World Health Organization (WHO) recommends exclusive breastfeeding for the first 6 months of an infant’s life followed by the addition of nutritionally adequate and safe complementary foods while breastfeeding continues for up to 2 years of age or beyond ^3^. In Australia, there is a high rate of lactation initiation (96%). However, these figures decline rapidly after birth, and only 15.4% of infants are exclusively breastfed at 5 months of age ^4^. Thus, in Australia, as in many other high-income countries, almost all mothers elect to breastfeed, but many encounter difficulties that affect their ability to continue.

Vital to the assessment of function of major organs is the objective measurement of various aspects of their physiology and biochemistry and the comparison of these values with reference ranges. An objective measurement of normal function can then form the scientific basis for identification of potential abnormality (function falling outside the normal range), diagnosis of disease, and the evaluation of treatments. Unfortunately, there are no routine clinical tests available for the assessment of normal function for lactation.

## Normal human lactation

In order to identify objective measurements for normal lactation function, it is first necessary to define normal lactation. Here, “normality” is considered in the biological sense, namely that normal function occurs naturally and not as a result of any disease, treatment, or genetic abnormality. Normal function does not require medical intervention or support. Thus, the following definition for lactation during the first 6 months after a term birth is proposed.

Normal human lactation

- is comfortable for both the mother and infant
- provides adequate milk for the infant’s optimal growth and development
- requires coordinated maternal and infant adaptation that is facilitated by good maternal and infant health.

## Reference ranges and reference limits

In the context of biological function, a reference range describes a range of values for a physiological measurement in a healthy person. A reference range for a particular measurement is the interval between which 95% of values for a reference group taken from the general population fall. Values outside the upper and lower limits of a reference range are not necessarily abnormal but can be considered to be indicators of possible pathology. A reference range is derived from a normal curve based on measurements for at least 120 healthy individuals. A reference limit describes cases where only one side of the range is of interest ^15^.

The above definition of normal human lactation defines the inclusive and exclusive parameters for studies aimed at determining reference ranges or reference limits (or both) for the assessment of human lactation. With the exception of the growth rate of breastfed infants (WHO growth charts ^16^), there are no studies that meet these criteria. Most substrates, metabolites, and hormones associated with human lactation change markedly according to the period of lactation, time of day, time of breastfeeding, duration of the breastfeed, and the degree of fullness of the breast. Thus, these factors need to be standardized for the development of normal ranges across the course of lactation. These deficiencies notwithstanding, it is nevertheless important to review the literature and construct approximate objective parameters for human lactation.

This review concentrates on areas where even roughly defined reference ranges could greatly assist in the understanding of lactation function (and consequently dysfunction). In this context, maternal parameters associated with secretory activation ^17^ and established lactation will be highlighted. In addition to this restriction to a consideration of maternal factors in the first instance, a pragmatic approach was taken to emphasize methods that could be implemented now (for example, blood progesterone and milk sodium) and then methods that could be readily developed in the future for clinical use (for example, milk lactose and milk production). There are many other factors that potentially could be used to assist clinical diagnosis, including certain proteins (α-lactalbumin), carbohydrates (oligosaccharides), lipids (medium-chain fatty acids), and enzymes (aurora kinase-A), but there is not enough evidence in human lactation to provide potential diagnosis and treatment protocols.

## Lactation cycle

The breast reaches a mature functional state only during lactation ^18^. The lactation cycle begins with conception. Pregnancy induces ductal proliferation and subsequent lobular alveolar development in the mammary gland. Alveolar development leads to secretory differentiation ^17^ with the maturation of lactocytes and the production of unique milk components. Delivery of the placenta triggers secretory activation and the transition to copious milk secretion. Sustained milk synthesis requires the continuation of efficient and regular milk removal by the infant in the context of normal function or by breast pump or hand expression if required. The lactation cycle is completed when the breast returns to quiescence following weaning of the infant.

## Secretory differentiation and secretory activation

Lactation is established in two phases. First, secretory differentiation is observed as the breast develops the capacity to synthesize unique milk products in colostrum, including lactose, casein, α-lactalbumin, and lactoferrin. The second phase, secretory activation, begins around 60 hours (range of 24–72 hours) after birth and is triggered by delivery of the placenta ^19, 20^.

## Secretory differentiation

There is little information on variation in breast growth and function during pregnancy. Breast volume generally increases after conception, but there is considerable variation between mothers ^21^. Importantly, this variation does not appear to influence the potential for milk synthesis postpartum ^5, 19, 22^.

Secretory differentiation occurs from about 20 weeks of pregnancy and requires the action of a lactogenic complex of hormones. The increasing concentration of prolactin in maternal blood is related to the increased excretion of lactose in urine, and the increase in the concentration of human placental lactogen is related to breast growth. During this phase, the tight junctions between lactocytes are leaky, allowing milk constituents to pass into the blood. Lactose cannot be metabolized once it enters the blood vascular system and is excreted in the urine. Therefore, the 24-hour output of lactose in urine provides a measure of the synthesis of lactose in the mammary glands. Coupling the 24-hour output of lactose in urine with the measurement of lactose in colostrum provides an estimate of the rate of synthesis of colostrum during pregnancy.

These measurements confirm that the rate of colostrum synthesis at this time is low (about 30 mL/24 hours) ^17, 23^.

## Secretory activation

**Figure 2.**
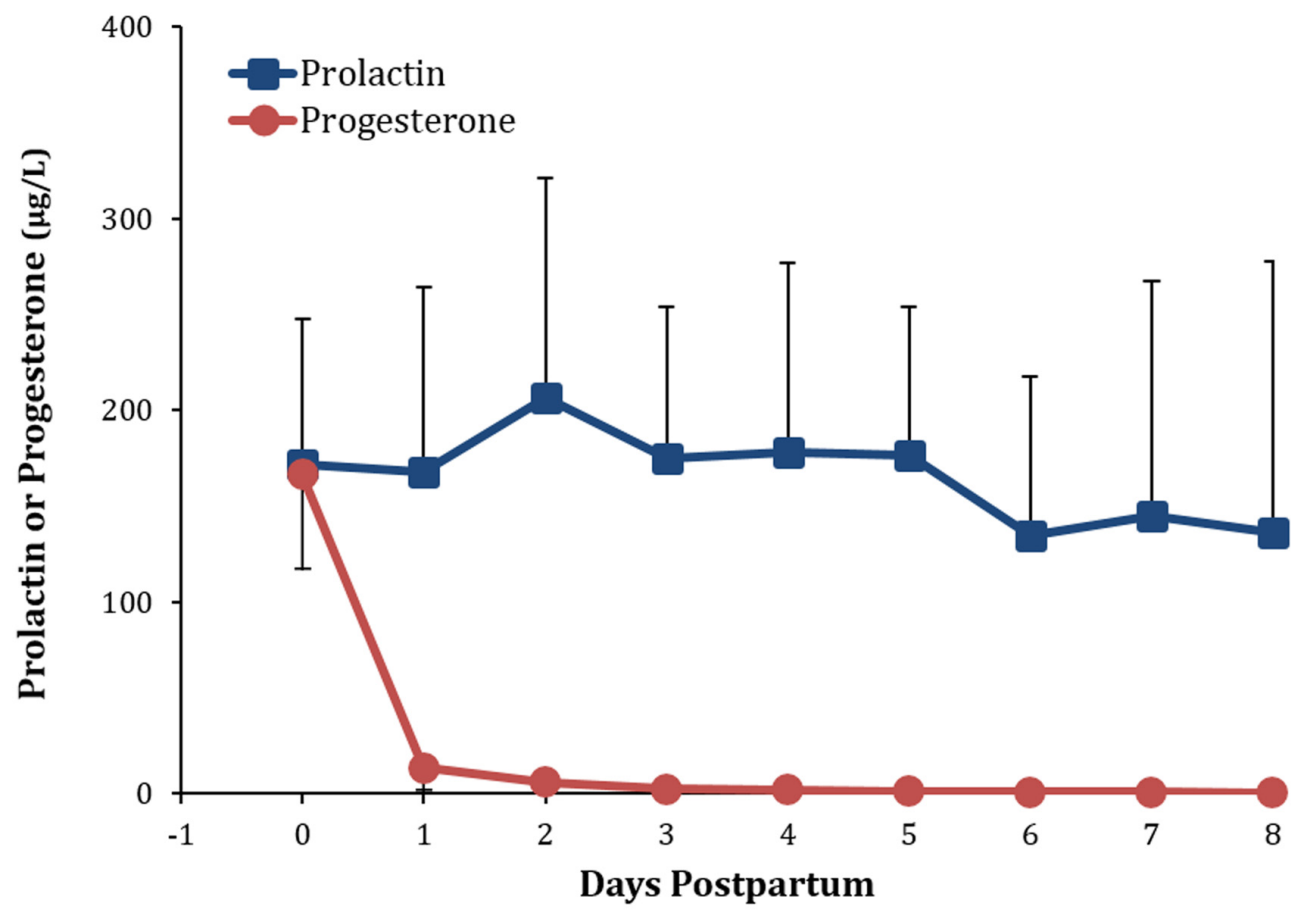
Daily concentrations of serum progesterone and prolactin in the first 8 days postpartum. Figure shows weighted means and standard deviations collated from published values. Data sources: 24– 31.

**Figure 3.**
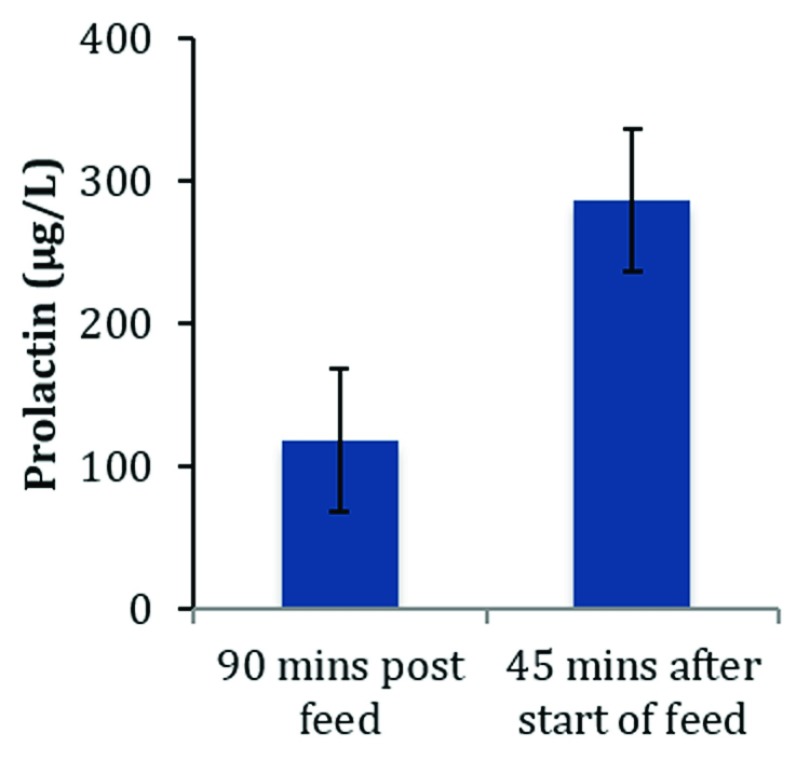
Serum prolactin levels 90 minutes post-feed and 45 minutes after beginning of feed at 1 month postpartum. Data source: 32.

**Figure 4.**
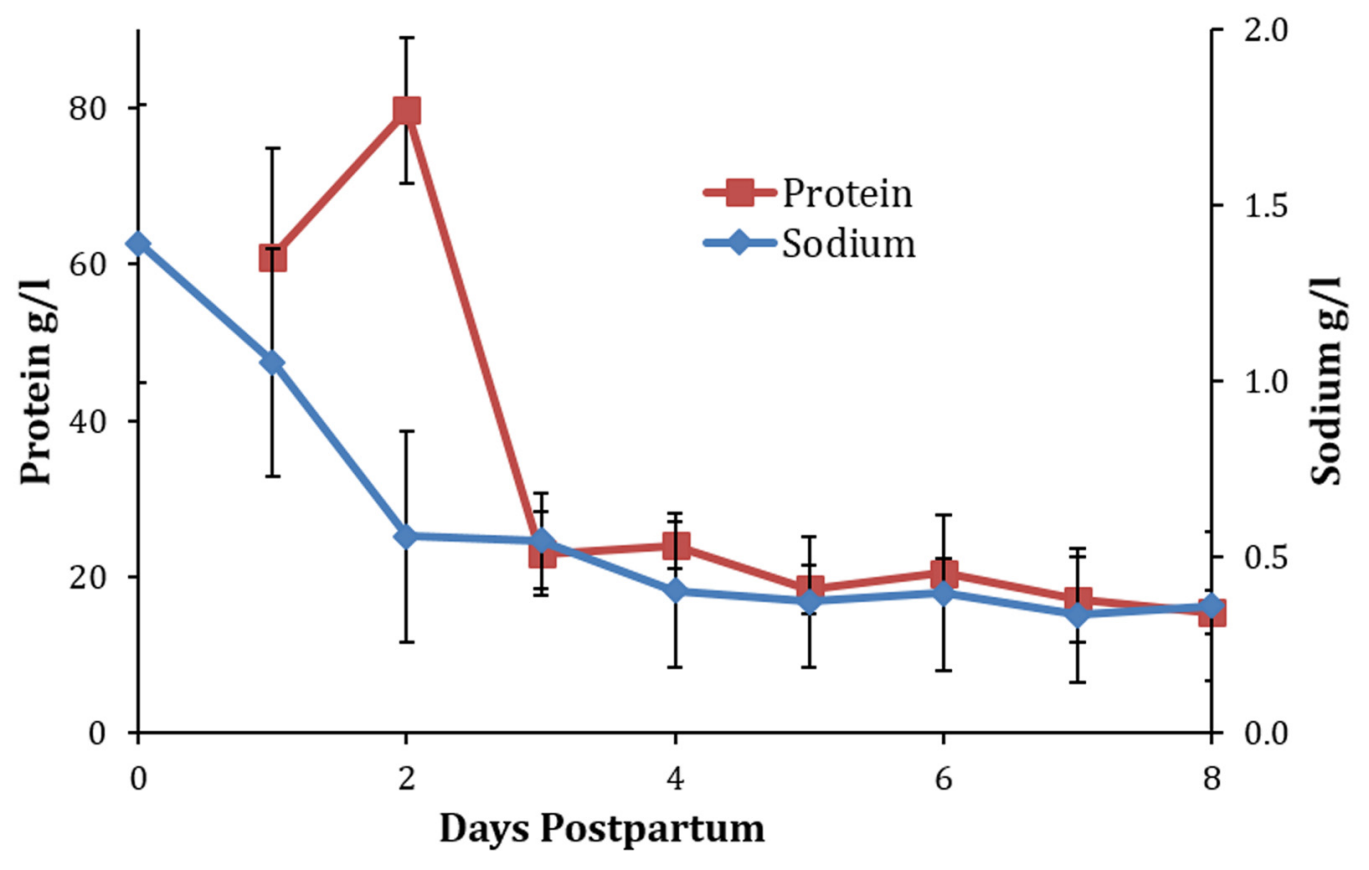
Daily breastmilk concentrations of total protein and sodium in the first 8 days postpartum. Figure shows weighted means and standard deviations collated from published values. Data sources: 7, 36– 50.

Secretory activation is a process manifest by the initiation of copious milk secretion ( Figure 1). The increase in milk secretion is accompanied by many metabolic changes. The most documented of these changes are decreases in blood progesterone ( Figure 2), milk protein, and sodium ( Figure 3) and increases in milk lactose and citrate ( Figure 4).

### Colostrum and the first 3 days

Colostrum is available to the infant for the first 60 hours (range of 24–72 hours) after birth ^19, 20^. The volume ingested by healthy newborns in the first 24 hours of life is small and mirrors synthesis (29 mL ± 24, mean ± standard deviation [SD]) ( Figure 1). Research interest in colostrum is limited for two reasons: first, from the generalized lack of research into human lactation (see above) and, second, by its appearance and small volume. More than 300 years ago, Cadogan observed that, “When a child is first born, there seems to be no provision at all made for it: for mother’s milk seldom comes in ’till the third day: so that according to nature, a child would be left a day and a half or two days, without food; to me a very sufficient proof that it wants none” ^33^. Furthermore, Dettwyler reported that, “In historical times, and even today, babies in some societies are denied colostrum, with all its beneficial properties, in the belief that it is a poisonous substance dangerous for the newborn” ^34^.

In contrast to historical beliefs, evidence is now emerging that the first 3 days postpartum are critically important for the establishment of lactation. In a study of pump-dependent mothers who had delivered preterm, Meier *et al*. compared milk expression by using the standard suction pattern on a breast pump with using a pattern that mimicked the infant sucking ^35^. The pattern was applied from birth to 3 days postpartum and then a standard pump was used up to 14 days postpartum. This treatment resulted in a 60% increase in milk synthesis from 6 to 14 days postpartum compared with the standard pumping pattern. Morton *et al*. studied pump-dependent mothers who had delivered preterm and combined hand massage with electric pumping in the immediate postpartum period and found that this intervention increased milk synthesis at 2 weeks compared with the use of a standard breast pump from birth ^51^. Additionally, a study in the Democratic Republic of the Congo reported that 14% of mothers provided with standard care and 16% provided with the Ten Steps to Successful Breastfeeding program were exclusively breastfeeding at 6 months postpartum but that 45% of mothers provided with only steps 1 to 9 were exclusively breastfeeding at 6 months ^52, 53^. These findings show that subtle interventions in the early postpartum period can induce profound outcomes during established lactation.

### Progesterone withdrawal

Kuhn first demonstrated that the withdrawal of progesterone was related to the ovary switching from progesterone to 20αOH-progesterone synthesis ^54^. The resulting fall in progesterone was shown to be the “lactogenic trigger” in the rat ^54^. Subsequently, in all mammals studied, progesterone withdrawal has been found to be the trigger for secretory activation. In women, progesterone is synthesized in the placenta and falls precipitously by more than an order of magnitude after the delivery of the placenta ( Figure 2). Consequently, colostrum synthesis continues until about 24 to 72 hours after birth when the process of secretory activation initiates the transition of colostrum into mature breastmilk. Thus, if viable fragments of placenta are retained after delivery, secretory activation will be either fully or partly inhibited.

Assays for progesterone concentration in blood are readily available. In addition, laboratory assays for progesterone concentrations in milk are available, and an in-home assay for urinary metabolites of progesterone has been developed ^55^. Despite the availability of these assays, progesterone is never measured when assessing the initiation of lactation in women who are at risk of or appear to have impaired secretory activation.

Given what is known about progesterone withdrawal triggering secretory activation, it can be hypothesized that elevated blood progesterone is a sensitive indicator of retained viable placental fragments. Furthermore, it is possible that the inhibitory effects of elevated blood progesterone on secretory activation are reduced by the administration of mifepristone (RU486), a progesterone antagonist. However, more research is required before these possibilities can be adopted clinically.

### Prolactin

At secretory activation, elevated concentrations of prolactin are observed during the period of progesterone withdrawal ( Figure 2). Plasma prolactin is high early in lactation and progressively decreases, but levels at 6 months of lactation are still higher than those reported for non-lactating women ^32^. The role of prolactin during the initiation of lactation is complex ^18^. The concentrations reported for prolactin during the early postpartum period are highly variable and difficult to interpret. This can be attributed to the secretion of prolactin varying in response to the suckling stimulus, and peak values are observed about 45 minutes after the infant latches to the breast ( Figure 3). Levels then decrease to about half the peak levels by the next breastfeed. Prolactin also shows a circadian variation ^56^ and increases at mealtimes. No studies have monitored prolactin levels in relation to the timing of breastfeeds, meals, and time of day, and this probably accounts for the large variation in reported concentrations of prolactin during secretory activation. Prolactin is present as different isoforms and undergoes considerable post-translational modifications, including glycosylation, phosphorylation, proteolytic cleavage, and polymerization. These modifications influence the function of this complex hormone. It is clear that prolactin is required for successful secretory activation ^57^, but it probably does not play a rate-limiting role.

The threshold concentration of prolactin (reference limit for normality) needed for normal secretory activation is unknown. This information is urgently required to ensure that mothers with normal concentrations of prolactin are not subjected to unwarranted medication with peripherally selective dopamine D _2_ receptor antagonist activity to treat low milk supply.

### Closure of tight junctions and the effect on milk composition

**Figure 5.**
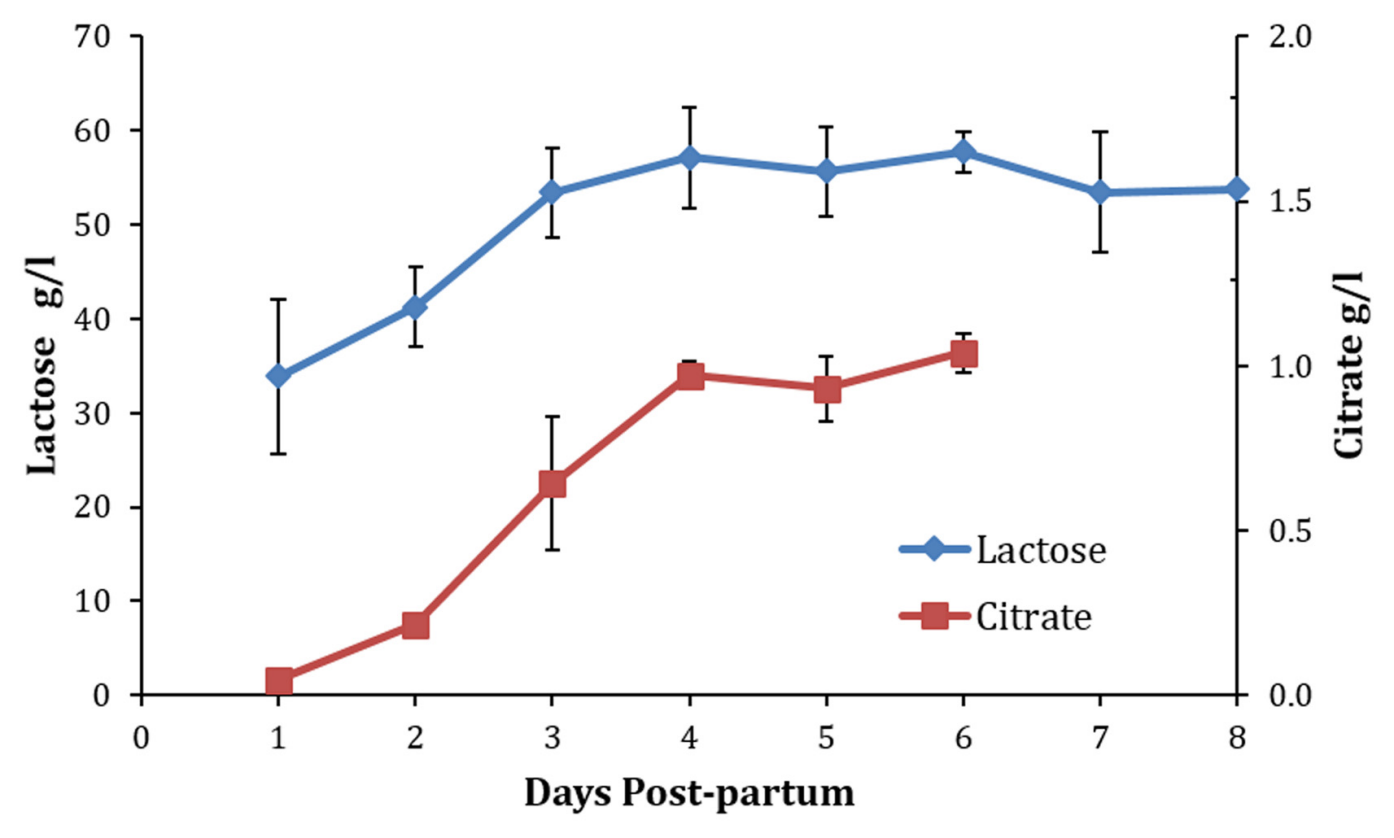
Daily breastmilk concentrations of lactose to day 8 and citrate to day 6 and citrate in the first 8 days postpartum. Figure shows weighted means and standard deviations collated from published values. Data sources: 7, 39, 42, 43, 45, 47– 49, 65– 70.

Closure of the tight junctions between lactocytes is related to the withdrawal of progesterone. At this time, the concentration of sodium in the mammary secretion rapidly decreases ( Figure 5) as milk production increases ( Figure 1) and provides a simple objective assessment of the progress of secretory activation ( Figure 4) ^58^. Indeed, sodium-sensitive electrodes that permit the measurement of sodium using only a few drops of breastmilk are available (Na ^+^ LAQUA twin electrode, Horiba Scientific, Kyoto, Japan).

Milk volume and the concentrations of sodium, lactose, citrate, and total protein all appear to reach stable levels by 8 days postpartum ( Figure 1, Figure 4, and Figure 5).

Decreases in sodium and total protein and increases in lactose and citrate in the mammary secretion can be used to monitor secretory activation ^5, 59^ ( Figure 4 and Figure 5). Collation of the published values for the changes in progesterone, sodium, total protein, lactose, and citrate enables the calculation of weighted daily means. From these means and SDs, it is possible to calculate reference limits for normality ( Table 1). It follows that the mean plus two SDs for progesterone in maternal blood and sodium and total protein in milk as well as the mean minus two SDs for lactose and citrate in milk provide reference limits that could be considered normal. Reference limits for days 3 and 6 are presented in Table 1. However, it must be recognized that these are tentative values because very few studies have determined the changes in blood progesterone and milk composition in the same mothers and, more importantly, most studies do not provide an objective assessment of the success of the mothers in establishing normal lactation.

**Table 1.** Reference limits at 3 and 6 days postpartum *.

	3 days postpartum	6 days postpartum
	Maternal serum	Maternal serum
Progesterone, μg/L	<7.1	<2.2
	Milk	Milk
Total protein, g/L	<34.1	<24.3
Sodium, g/L	<0.81	<0.84
Lactose, g/L	>43.4	>53.6
Citrate, g/L	>0.24	>0.92

*Expressed as weighted means ± two standard deviations from the mean for maternal serum concentrations for progesterone and milk concentrations for sodium, total protein, lactose, and citrate

As stated, milk sodium can be measured to track the progress of secretory activation. In the future, timed urinary lactose measurements after birth would also be useful. High levels of urinary lactose combined with low levels of milk sodium would indicate that lactose synthesis (milk synthesis) is occurring but the milk is not being removed from the mammary gland; that is, there is a “baby” problem rather than a “mother” problem.

Citrate has been claimed to be the harbinger of lactation ^60^, but the metabolic stimulus driving the increase of this metabolite is not known. Citrate still provides a very good marker for secretory activation ( Figure 5). It is not measured routinely in clinical pathology laboratories and therefore is not available at this time for the assessment of secretory activation.

## Milk synthesis and milk production

It is important to define the difference between milk synthesis and milk production measurements. Whereas milk synthesis is a measure of the maternal capacity to synthesize milk, milk production is a measure of the infant’s ability to remove milk from the mother’s breasts ( Figure 1). Daily progressive measurements of both of these parameters would be very useful in the assessment of lactation ^6, 7^. Twenty-four-hour measurements of milk production by weighing the infant immediately before and after each breastfeed to determine the milk intake is extremely difficult to carry out in the immediate postpartum period. Milk synthesis cannot currently be measured directly; however, the increase in milk citrate appears to be closely related to the mother’s ability to synthesize milk. A greater knowledge of the metabolic factors related to the increase in citrate in early postpartum milk secretion is required to confirm this possibility.

## Established lactation

Once lactation is established from about 2 weeks postpartum, milk production remains relatively constant up to 6 months of lactation for infants that are exclusively breastfed ^1^. Milk synthesis is not limited by the capacity of the mother to synthesize milk but rather by the infant’s appetite ^67^. Thus, milk production in breastfeeding mothers is not a measure of the mother’s capacity to synthesize breastmilk but rather a measurement of the infant’s appetite. Furthermore, the variation in milk production reported between infants is large, ranging from about 500 to 1,000 mL/24 hours with a mean of 750–800 mL/24 hours ^1^. The WHO Child Growth Standards provide reference values for breastfed infants; however, nearly all studies of normal intake of exclusively breastfeeding infants precede their release. Therefore, reference values for breastmilk production require further investigation. Nevertheless, measurement of milk production is currently the best objective method available to measure normal breast function during established lactation.

### Measurement of milk production

The most clinically feasible measure of breastmilk transfer to the infant is to weigh the infant immediately before and after each breastfeed over a 24-hour period ^67^. From a nutritional standpoint, a longer period would be desirable; however, this method is quite demanding of mother and infant and the quality of the data degrades as the collection period is extended. Alternatively, each breast can be expressed for 10 minutes every hour for three consecutive hours in a calm environment. The volume of milk expressed at the third expression multiplied by 24 also provides an estimate of 24-hour milk synthesis ^67^. It is possible to use this method in a clinical setting, but further validation is required. Total breastmilk transfer from both breasts over a period of 14 days can be measured by using the deuterium oxide dose-to-mother technique. This is an excellent technique for nutritional studies for determining macro- and micro-nutrient intake. However, this method is based on the dilution of the isotope over a period of 14 days and requires stable milk production over this period. Measurement of milk production is a good starting point for assessment of breast function, particularly when there are concerns that supply is inadequate.

### Milk fat

Fat is the most variable component of human milk, and around 70% of this variation is due to the extent of breast fullness ^68^. As the breast empties, the fat content increases ^68^. This provides one of the few objective tests available to determine the proportion of available milk removed by the infant during a breastfeed. Mature milk from a full breast appears bluish-white because of the low fat content pre-feed. Post-feed, the milk changes in color to creamy white as the breast is drained and the fat content increases. This response is more obvious if the milk is allowed to settle. If there is not much change in the color of the milk between the pre-feed and post-feed samples, the baby has not removed much milk. Furthermore, if the pre-feed milk is bluish-white, the breast is full of milk, and if the pre-feed milk is creamy white, the breast is drained of milk. Because visual assessments of a breastfeed are very unreliable, observations of the color of the milk can provide only limited objectivity in the clinical assessment of a breastfeed. A more objective measure of breastmilk transfer was discussed previously. The change in fat content can be measured objectively with a creamatocrit centrifuge (Medela AG, Baar, Switzerland) ( Table 2) ^69^.

**Table 2.** Concentrations of fat in mature human milk ^1, 19^.

Fat	Amount, g/L	Standard deviation, g/L
Average	41.1	± 7.8
Pre-feed	30.3	± 17.5
Post-feed	57.8	± 24.0

### Milk ejection

Milk ejection occurs because of a neuro-hormonal reflex triggered by stimulation of the nipple areolar area, which results in the contraction of myoepithelial cells surrounding the alveoli and subsequent expulsion of milk. It is a conditioned reflex and is responsive to environmental inputs. Each woman has multiple milk ejections during a breastfeed, but most mothers sense only the first. Milk ejection can be readily measured by ultrasound imaging of the non-suckled breast ^70^. In mothers who do not sense milk ejection, measurement of an increase in duct diameter during a breastfeed can be used to confirm milk ejection.

## Conclusions

In a whole of body comparison, the lactating human breast rivals the brain in its energy requirements and has the highest energy requirement of the organs in the reproductive cycle. Evidence-based reference ranges or limits for normal function (or both) provide a foundation for clinical diagnosis and treatment of problems encountered. Despite its metabolic importance, there is an enormous gap in our understanding of these parameters for the biochemistry and physiology of lactation. This review has shown that only approximate reference ranges can be calculated. Nevertheless, they provide a starting point.

There have been many successful health programs aimed at encouraging women to breastfeed their babies, but there is little ability to adequately monitor and support lactation initiation and establishment with objective tests. Under these conditions, it is not surprising that in high-income countries, even when most women choose to breastfeed, the rate of sustained lactation rapidly declines after birth. It seems obvious that to effectively “close the gap” more scientific evidence must be integrated with psychological and practical support for all women who want to breastfeed their infants.
